# Effects of a shipwreck on the zooplankton community in a port region of the Amazon

**DOI:** 10.1007/s11356-018-3999-2

**Published:** 2019-01-05

**Authors:** Samara Pinheiro, Marcelo Lima, Bruno Carneiro, Vanessa Costa Tavares, Volney Câmara

**Affiliations:** 1 Institute of Studies in Collective Health, Federal University of Rio de Janeiro (IESC/UFRJ), Rio de Janeiro, Brazil; 2 Environmental Section, Evandro Chagas Institute (SAMAM/IEC), Ananindeua, Brazil; 3 Laboratory of Environmental Biology/Environmental Section, Evandro Chagas Institute (SAMAM/IEC/SVS/MS), Rodovia BR-316, Km 07, s/n, Levilândia, Ananindeua, PA CEP 67030-070 Brazil

**Keywords:** Shipwreck, Zooplankton, Environmental indicators, Aquatic ecosystems, Environmental monitoring, Amazon region

## Abstract

The port regions of the Amazon are subject to environmental impacts high shipping traffic. In October 2015, a cargo ship containing 5000 oxen sank in the Port of Vila do Conde, northern Brazil, releasing large amounts of organic matter and diesel oil into the aquatic environment. We evaluated the consequences of this shipwreck on the zooplankton community. Sampling was carried out using a phytoplankton net (64 μm) at two locations close to the port. We calculated the frequency of occurrence, relative abundance, and trophic state index and performed a canonical redundancy analysis of zooplankton in this area. Total density values ranged from 371 to 8600 organisms/m^3^, with minimum values being recorded during the period of the shipwreck and maximum values after the shipwreck. The areas categorized as super eutrophic had the lowest density values. The most abundant species/groups were nauplii and copepodites of the orders Cyclopoida and Calanoida. Of the environmental variables, only biochemical oxygen demand, chemical oxygen demand, and total dissolved solids were selected by the redundancy canonical analysis. The environmental conditions of the region and the ongoing environmental impacts might substantially influence the structure of the zooplankton community. The predominance of these organisms, in addition to the high densities of nauplii and copepodites, was likely related to the large amounts of nutrients generated by the shipwreck.

## Introduction

Most of the rivers in the Amazon region are navigable via the 24,000-km network of waterway systems. Within this network, there are small, medium, and large ports that have significant importance for local and global economies, owing to their involvement in the transport of agricultural, mineral, and industrial materials (Hofmann 2015; Sant’anna 1998). However, these ports also have environmental impacts that considerably change the quality of the surface water and affect aquatic ecosystems.

The port and industrial areas in the city of Barcarena are subject to environmental damage because of intense shipping traffic, fuel spills, the release of chemical substances, and the introduction of harmful organisms (Paz et al. 2011), all of which pose risks to human health (Porto and Teixeira 2002). The Port of Vila do Conde is considered the largest port terminal in the Amazon. It is located in the coastal zone of the Central Amazon basin, on the banks of the River Pará in the City of Barcarena, Northern Brazil. In the 1980s, government incentives attracted large companies that operate to extract and refine ores, such as kaolinite and bauxite, to this area (Paz et al. 2011).

On average, 32 ships per month pass through this port, transporting raw material (e.g., bauxite, alumina), refined mineral cargo (e.g., aluminum ingots), other materials (e.g., fuel oil, tar, coke, caustic soda), and general cargo (e.g., timber, live cattle, fertilizers, containers) (Rodrigues and Szlafstein 2013). The export of live cattle has increased significantly in the last three decades, and a large portion of total production is shipped through the Port of Vila do Conde.

The present study highlights the sinking of a ship that was carrying approximately 5000 oxen in this region in October 2015 (hereafter referred to as “the shipwreck”). Large quantities of animal tissue and diesel oil were released into the river. The hydrological dynamics of this region further contributed to the spreading of these materials over a large area. The spill reached the islands and beaches of Barcarena and other nearby cities, causing serious environmental, social, and economic problems. It compromised the integrity of the aquatic ecosystems and damaged quality of life in the riverside populations of the region (IEC-SEMAN 2015a).

Many biological communities were affected by this shipwreck, including plankton, which form the foundation of the aquatic food chain and are highly sensitive to environmental disturbances. The zooplankton community is an indicator of the trophic conditions in a given area. Because zooplankton has a short life cycle and their population dynamics are strongly related to physical and chemical variability of the water, they quickly reflect changes in the aquatic environment (Cairns et al. 1993).

Zooplanktonic organisms are the key links in the transfer of energy along the food chain in aquatic environments. Therefore, they are useful bioindicators in studies that aim to evaluate environmental impacts and risks to human health, by investigating their biological characteristics and contributions in pelagic ecosystems (Pinto-Coelho et al. 2005; Silva 2011).

Knowledge about the population structure of zooplankton and seasonal variation in zooplankton species provides relevant information about the potential organic production of a given area (Eskinazi-Sant’anna et al. 2013; Sampaio et al. 2002). The composition and abundance of zooplankton species might be influenced by a number of physical, chemical, and biological factors, such as temperature, salinity, pH, quality and availability of food, competition, ecology, and predators (Park and Marshall 2000). In natural environments, these factors act simultaneously and might interact synergistically to modify the structure of zooplankton communities, leading to the disappearance and/or proliferation of certain species (Sládeck 1983).

The present study aimed to evaluate the immediate consequences of a shipwreck in the largest port area of the Amazon region. The shipwreck caused changes to various environmental processes, which might cause significant changes to water quality and, consequently, the zooplankton community.

## Material and methods

### Study area

This study was conducted in the Amazon port region, which encompasses the territorial areas of the city of Barcarena, State of Pará, Brazil. The Port of Vila Conde is located on the right margin of the Pará River and is considered the largest port terminal in the Amazon.

According to the classification of Köppen, the climate of this region is classified as an Am type, characterized as hot and humid with high temperatures (annual average, 27 °C) and high precipitation. The average precipitation exceeds 2500 mm per year, reaching up to 400 mm per month in the rainy period and less than 30 mm per month in the driest months.

**Fig. 1:**
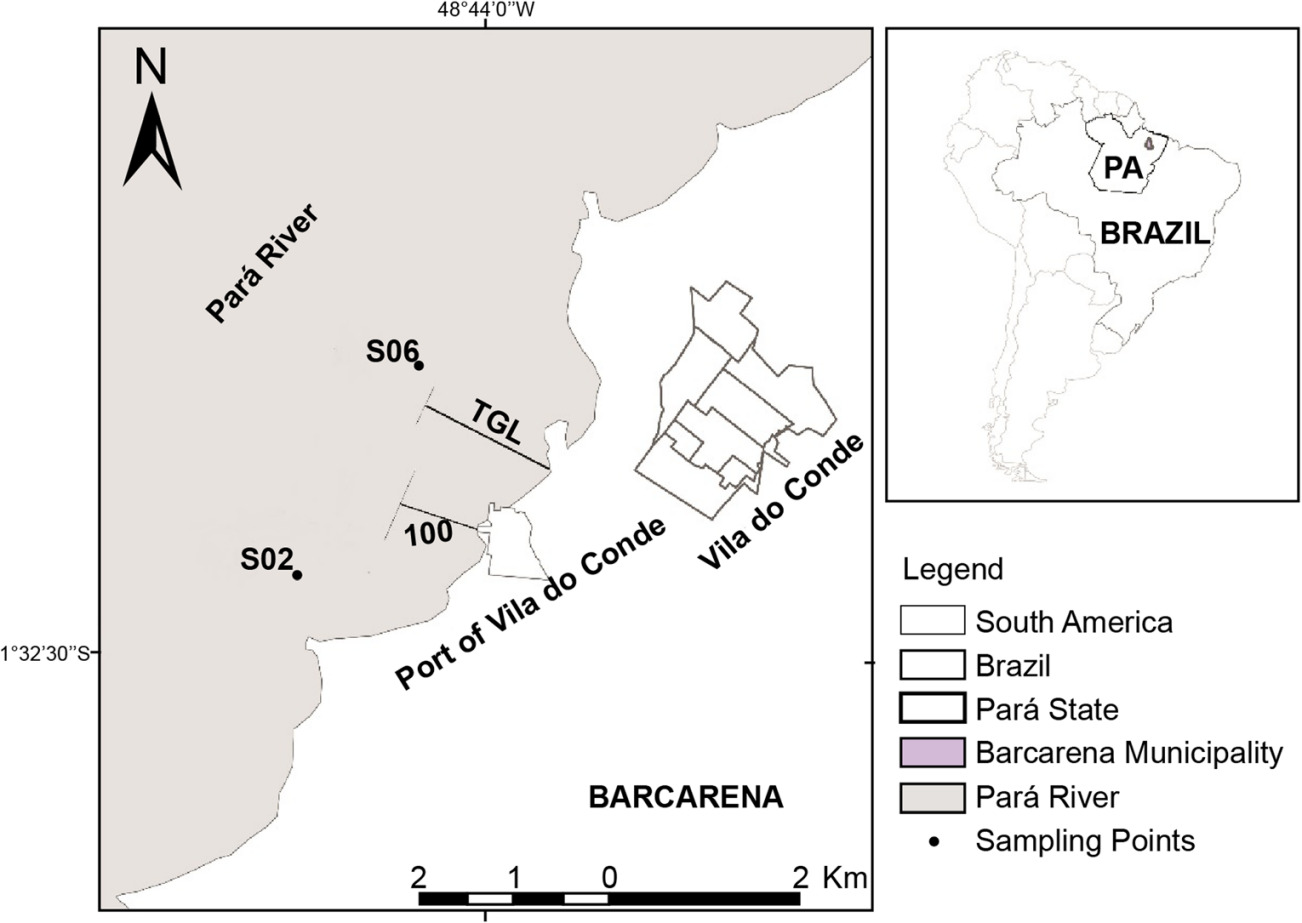
Location and sampling points in the Port of Vila do Conde, State of Pará, Brazil

These ecosystems are continuously impacted by the industrial complex located in this region (Piratoba et al. 2017). Agriculture, artisanal fisheries, and industry are the main activities conducted in this region. In particular, many companies have been producing aluminum since the 1980s, with this industry still growing due to mineral-metallurgical activities and the port location (Paz et al. 2011). For the purposes of this study, two sampling sites were established in the Port of Vila do Conde: S02 (upstream point of the port) and S06 (downstream point of the port) (Fig. 1).

### Shipwreck

On October 6, 2015, a cargo ship carrying 5000 oxen for export sank in the Port of Vila do Conde. Large amounts of biological material (carcasses, bones, and fluids), as well as fuel (approximately 700,000 L of diesel oil), spilled into the River Pará. Consequently, the islands and beaches of this region were closed for a number of weeks (IEC-SEMAM 2015a).

### Sampling

Water samples were collected to analyze water quality on October 8 and October 15, 2015. To assess the impacts of the shipwreck on the zooplankton community, we compared the data collected on October 8 and 15 with data collected in September 2015. We also compared the data collected in September 2015 (before sinking) to that collected in November 2015, directly after the sinking. We also compared our results with previous (unpublished data) environmental monitoring data collected in the study region in September and December of 2014 (IEC-SEMAM 2015b).

Samples were collected during periods of tidal flood and ebb; however, at the time of the shipwreck, sampling was only carried out during the ebb tide. For limnological evaluations and the determination of the concentrations of chlorophyll-a, we used 500-mL polypropylene bottles. Zooplankton were sampled using plankton nets (64 μm) equipped with flowmeters (General Oceanics Inc., Florida, EUA) by performing horizontal trawling below the surface of the water column during both sampling seasons (S02 and S06). After collection, all materials were fixed in a 4% formalin solution and placed in 250-mL polypropylene vials.

### Physiochemical and biological analyses

#### Limnological variables

We measured the following physicochemical water parameters: temperature (T) in °C, hydrogenionic potential (pH), electric conductivity (EC), total dissolved solids (TDS), salinity, and dissolved oxygen (DO), using a portable multiparameter meter (HI9828 HANNA®). The following variables were determined by UV-VIS spectrophotometry: turbidity (Turb.), total suspended solids (STS), and chemical oxygen demand (COD). To measure the biochemical oxygen demand (BOD), samples were incubated over a period of 5 days (APHA 2005). Concentrations of the ions N-nitrite (N-NO_2_^−^), nitrate-nitrogen (N-NO_3_^−^), nitrogen ammonium (N-NH_4_^+^), and total phosphorus (P_total_) were measured via ion chromatography (ICS DUAL 2000-DIONEX). To analyze chlorophyll-a, the samples were filtered through glass fiber filters (GF-3, 47 mm, Millipore) using a vacuum pump (EMD, Millipore). Chlorophyll-a concentrations were calculated according to Strickland and Parsons (1972).

#### Trophic state index

The trophic state index (TSI) was calculated based on the trophic state index for phosphorus—TSI (PT)—and the trophic state index for chlorophyll a—TSI (CL)—modified by Lamparelli (2004). The calculation performed generates a numeric index that classifies the water as ultraoligotrophic (TSI = 47), oligotrophic (47 < IET ≤ 52), mesotrophic (52 < IET ≤ 59), eutrophic (59 < IET ≤ 63), supereutrophic (63 < IET ≤ 67), or hypereutrophic (IET > 67).

#### Zooplankton

A qualitative zooplankton analysis was carried out on subsamples in petri dishes using 4 mL water (stained with 1% rose bengal) from each sampling station (CETESB 2000). For the quantitative analysis, we prepared three sub-samples (3 mL total) in a Sedgewick Rafter (1 mL) counting chamber for observation under an inverted microscope (Axiovert A1, Carl Zeiss). Zooplankton were identified based on the published literature. We calculated the frequency of occurrence (FR) of species, the total density of zooplankton organisms (org/m^3^), and the relative abundance (RA) of each species. According to the FR, the species/organisms were classified as very frequent (present > 70% of the time), frequent (40%), infrequent (10%), and sporadic (< 10%) (CETESB 1978).

### Statistical analyses

We used a factorial ANOVA to compare environmental variables and biological parameters between stations and sampling periods. Statistical significance was determined at *p* < 0.05. To evaluate water quality based on the measured variables, we used Pearson’s correlation. To evaluate the relationships between the distribution of the recorded species/groups and environmental data, we applied a redundancy canonical analysis (RDA). Explanatory variables were selected using the package CANOCO 4.5 (Legendre and Birks 2012; Legendre and Legendre 2012). Indicator species (IndVal) were analyzed to identify the species/groups that were typical for each sampling period. These calculations were performed using the software package PC-Ord 6.0 (Dufrene and Legendre 1997).

## Results

### Limnological variables

Table 1 shows the values of the main variables recorded during the study period in comparison with the standards established by the Brazilian Legislation for Fresh Water Class 2 (CONAMA 2005). During the shipwreck, the levels of DO, BOD, and total phosphorus exceeded threshold levels. The following variables showed significant differences between the different sampling periods: TDS (ANOVA, *F* = 37.3; *p* < 0.05), DO (ANOVA, *F* = 38.4; *p* < 0.05), Turb (ANOVA, *F* = 29.4; *p* < 0.05), Cla (ANOVA, *F* = 38.4; *p* < 0.05), BOD (ANOVA, *F* = 12.6; *p* < 0.05), DQO (ANOVA, *F* = 12.6; *p* < 0.05), NO_2_^−^ (ANOVA, *F* = 5.58; *p* < 0.05), and NO_3_^−^ (ANOVA, *F* = 38.4; *p* < 0.05).

**Table 1:** Limnological variables and trophic state index (TSI) recorded before, during, and after the shipwreck in the Port of Vila do Conde (State of Pará, Brazil)

Limnological variables	UNID	Before the shipwreck (September 2015)				Shipwreck period (October 2015)				After the shipwreck (November 2015)				Reference
		S2		S6		Day 08		Day 15		S2		S6		
						S2	S6	S2	S6					
		FT	ET	FT	ET	ET	ET	ET	ET	FT	ET	FT	ET	
pH	–	6.9	6.9	6.7	6.7	7.5	7.2	8.1	7.6	7.8	7.5	7.9	7.7	6–9
T	°C	29.7	29.2	29.2	29.0	29.9	29.7	29.6	29.3	28.8	28.6	28.8	28.7	Amb
EC	mS cm^−1^	92.6	90.7	92.7	91.4	54.3	60.0	116.0	39.0	61.0	62.0	42.0	54.0	–
TDS	mg L^−1^	58.1	56.1	56.8	55.2	54.3	60.0	58.0	19.0	31.0	31.0	21.0	27.0	–
Sal.	–	0.04	0.04	0.03	0.04	0.02	0.02	0.05	0.02	0.03	0.03	0.02	0.02	≤ 0.5
OD	mg L^−1^	6.74	6.69	6.72	6.2	3.9	6.07	8.11	7.92	8.76	8.15	8.53	8.14	≥ 5
Turb.	UNT	14.72	16.13	27.11	17.07	19.00	19.00	8.50	8.50	11.50	18.00	10.50	16.50	100
STS	mg L^−1^	6.5	9.5	8	8.5	12	13	3	4	5	9.5	5.5	9	–
Alca.	mg L^−1^	35	36	37	37	38	44	11	16	42	37	38	42	–
DBO	mg L^−1^	1.4	1.5	3.1	2.9	5.0	2.0	8.0	9.0	<LD	2.0	5.0	<LD	≤ 5
DQO	mg L^−1^	70	67	69	43	10	5	18	16	<LD	8	10	<LD	–
NO_2_^−^	mg L^−1^	0.005	0.013	0.008	0.005	0.004	0.001	0.003	0.002	0.006	0.006	0.007	0.007	1
NO_3_^−^	mg L^−1^	1.80	0.95	2.00	0.55	0.40	0.30	1.40	1.20	3.05	1.95	2.75	1.55	10
SO_4_^2−^	mg L^−1^	1	<LD	1	<LD	1	<LD	27	<LD	2	<LD	<LD	<LD	250
P_total_	mg L^−1^	0.09	0.17	0.07	0.11	0.02	1.89	0.07	0.08	0.06	0.04	0.07	0.07	0.05
NH_3_	mg L^−1^	0.375	0.250	0.220	0.210	0.170	0.310	0.160	0.060	0.090	0.110	0.055	0.220	–
Cla	μg L^−1^	6.84	8.41	6.62	4.07	2.35	3.28	3.30	9.20	3.16	2.79	17.76	0.73	30 μg/L
TSI	–	Eut	Super	Eut	Eut	Meso	Super	Meso	Eut	Meso	Meso	Super	Olig	–

*FT*, flood tide; *ET*, ebb tide; *LD*, quantification limit; *T*, temperature; *EC*, electric conductivity; *TDS*, total dissolved solids; *Sal*., salinity; *OD*, dissolved oxygen; *Turb*., turbidity; *STS*, total suspended solids; *Alca*., alkalinity; *DBO*, biochemical oxygen demand; *DQO*, chemical oxygen demand; *Cla*, chlorophyll-a; *TSI*, trophic state index; *Olig*, oligotrophic; *Meso*, mesotrophic; *Eut*, eutrophic; *Super*, supereutrophic

**Fig. 2:**
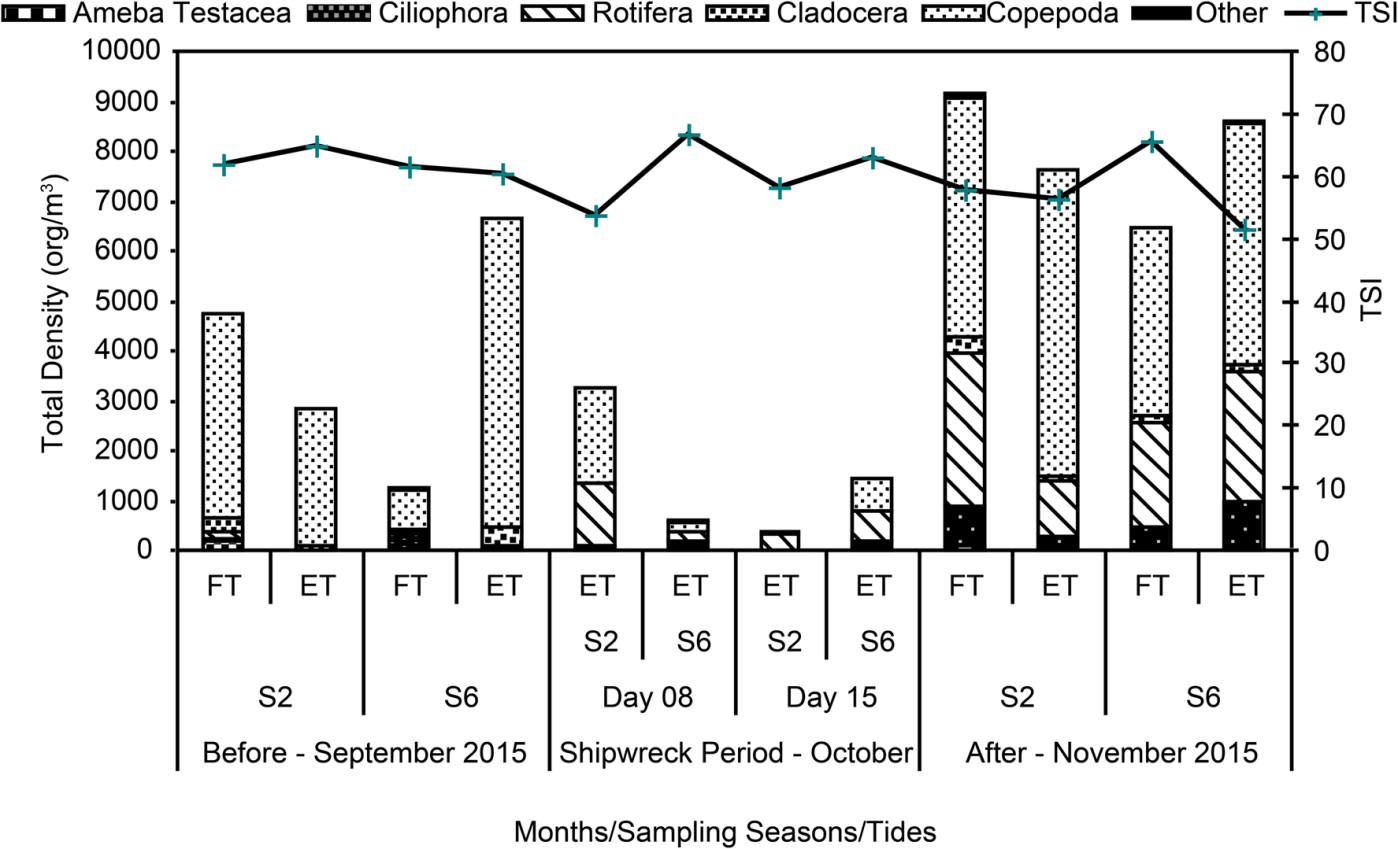
Total density of zooplankton (org/m^3^) and trophic state index (TSI) recorded before, during, and after the shipwreck at the Port of Vila do Conde, State of Pará, Brazil

Based on the TSI values, we classified the surface waters into different categories. The stations categorized as supereutrophic (63 < TSI ≤ 67) were S2 (September 2015, ebb tide), S6 (October 8, 2015), and S6 (December 2015; flood tide) (Table 1 and Fig. 2).

### Zooplankton

With regard to the frequency of occurrence, species/groups were classified as less frequent (65%), frequent (7%), and very frequent (28%).

The following species/groups were classified as very frequent during all of the analyzed periods: *Tintinnina* sp. 1, the nauplii of Copepoda, copepodites of Cyclopoida, *Filinia terminalis*, *Condonella* sp. 1, and *Keratella americana*. During the period immediately following the shipwreck, only three species were very frequent: *Lecane* species 1, *Trichocerca* sp. 1, and *Keratella cochlearis*. After the shipwreck, *Filinia opoliensis*, *Lecane proiecta*, *Brachionus caudatus*, *Moina minuta*, and *Diaphanosoma birgei* were the most frequent species (Table 2).

**Table 2:** Classification and frequency of occurrence (FR) of zooplankton taxa recorded before, during, and after the shipwreck in the Port of Vila do Conde (State of Pará, Brazil)

Taxa	Before (September 2015)	Shipwreck period (October 2015)	After (November 2015)
Phylum: Amoebozoa			
Order: Arcellinida			
Family: Difflugidae			
*Difflugia acuminata* Ehrenberg, 1838		IF	FR
*Difflugia corona* Wallich, 1864		IF	
*Difflugia elegans* Penard, 1890		FR	
*Difflugia* sp. 1	IF	IF	
*Difflugia* sp. 9	FR	FR	IF
Family: Centropyxidae			
*Centropyxis aculeata* (Ehrenberg, 1838) Stein, 1859			IF
Phylum: Ciliophora			
Order: Choreotrichida			
Suborder: Tintinnina			
*Tintinnina* sp. 1	VF	VF	VF
*Tintinnina* sp. 8			IF
Family: Codonellidae			
*Codonella *sp. 1	VF	VF	VF
Phylum: Arthropoda			
Nauplii of Copepoda	VF	VF	VF
Class: Malacostraca			
Ordem: Decapoda			
Protozoea		FR	IF
Class: Branchiopoda			
Neonates of cladocera	FR		IF
Order: Diplostraca			
Family: Chydoridae			
*Alonella dadayi* Birge, 1910			IF
Family: Sididae			
*Diaphanosoma birgei* Korinek, 1981	VF	IF	VF
Family: Moinidae			
*Moina minuta* Hansen, 1899	VF	FR	VF
Family: Bosminidae			
*Bosminopsis deitersi* Richard, 1895	FR		IF
*Bosmina hagmanni* Stingelin, 1904	VF	IF	
*Bosmina longirostris* Müller, 1776	VF		
Subclass: Copepoda			
Order: Calanoida			
Copepodites of Calanoida	VF	VF	VF
*Pseudodiaptomus* sp. 1	IF		
Order: Harpacticoida			
Harpactiocoida sp. 1			IF
Order: Cyclopoida			
Copepodites of Cyclopoida	VF	VF	VF
Cyclopoida species 1	VF	FR	VF
Class: Arachnida			
Subclass: Acari			
Acari		IF	
Phylum: Mollusca			
Class: Gastropoda			
Larvae of Polychaeta			IF
Larvae of Gastropoda	FR	FR	VF
Class: Bivalvia			
Larvae of Bivalve	IF	IF	
Phylum: Rotifera			
Rotifera sp. 1	FR		
Rotifera spp.		IF	
Order: Ploima			
Family: Brachionidae			
*Brachionus calyciflorus* Pallas, 1766		FR	FR
*Brachionus caudatus* Barrois and Daday, 1894	VF	IF	VF
*Brachionus falcatus* Zacharias, 1898			IF
*Brachionus mirus* f. *voigti* Koste, 1972ª	IF	FR	IF
*Brachionus urceolaris* Müller, 1773	VF		IF
*Brachionus zahniseri* f. *gessneri* Hauer, 1956	IF	FR	IF
*Brachionus zahniseri* f. *reductu* Hauer, 1956	IF	IF	
*Keratella americana* Carlin, 1943	VF	VF	VF
*Keratella cochlearis* Gosse,1851	FR	VF	VF
*Keratella lenzi* Hauer, 1953		IF	
Family: Lecanidae			
*Lecane proiecta* Hauer, 1956	FR		VF
*Lecane* sp. 1		VF	
*Monostyla* sp.		IF	IF
Family: Trichocercidae			
*Trichocerca elongata* Gosse, 1886		IF	
*Trichocerca pusila* Jennings, 1903		FR	IF
*Trichocerca* sp. 1	IF	VF	
*Trichocerca similis* (Wierzejski, 1893)	IF	IF	
*Trichocerca* sp.		FR	FR
Order: Gnesiotrocha			
Family: Filinidae			
*Filinia camasecla* Myers, 1938		IF	IF
*Filinia opoliensis* (Zacharias, 1898)	FR		VF
*Filinia terminalis* (Plate, 1886)	VF	VF	VF
Família: Hexarthridae			
*Hexartha* sp.	FR		
Order: Flosculariaceae			
Family: Testudinellidae			
*Testudinella patina* (Hermann, 1783)		IF	

*IF*, infrequent; *FR*, frequent; *VF*, very frequent

The total density of organisms ranged from 371 to 8600 org/m^3^, where minimum values were observed during the shipwreck and maximum values after the shipwreck. The maximum and minimum values were both recorded at site S2 (Fig. 2). There were significant differences between the density values and sampling periods (ANOVA, *F* = 38.4; *p* < 0.05) and stations (ANOVA, *F* = 40.3; *p* < 0.05). The high densities recorded at site S2 might be attributed to a greater concentration of organisms belonging to the subclass Copepoda (4823 org/m^3^) and the phylum Rotifera (2624 org/m^3^).

**Fig. 3:**
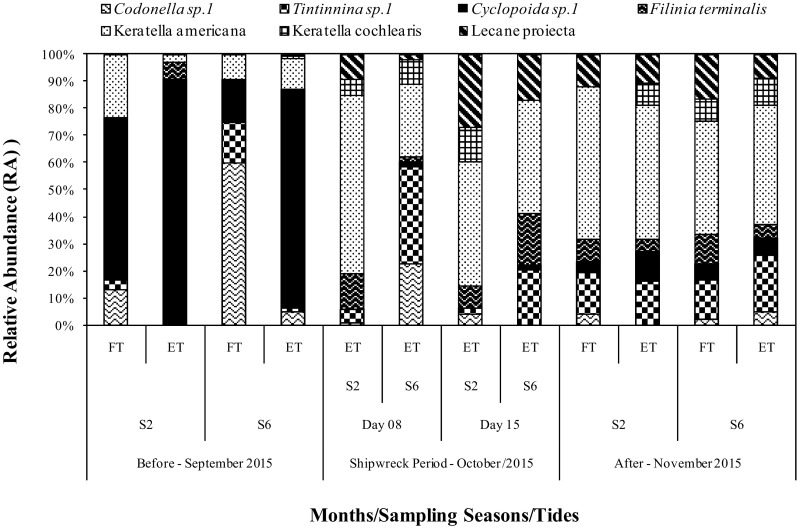
Relative abundance (RA) of the main species recorded, before, during and after the shipwreck in the Port of Vila do Conde, State of Pará, Brazil

During the shipwreck, the stations categorized as supereutrophic (S6, ebb tide, October 8 and 15) exhibited lower density values during the entire study period than those representing other sampling times and locations (Fig. 2). With regard to relative abundance (RA), we only considered the species/groups that presented values higher than 20%. The most representative species/groups were the nauplii and copepodites of Cyclopoida and Calanoida. When we removed these organisms from the community, the species with higher values of RA during the entire sampling period were *K*. *americana*, *Tintinnina* sp. 1, *Lecane* sp. 1, *F*. *terminalis*, *Codonella* sp. 1, Cyclopoida sp. 1, and *K*. *cochlearis* (Fig. 3).

### Interactions between limnological variables and zooplankton

Table 3 presents the main correlations between the limnological variables. Of note, several of the analyzed variables are negatively correlated.

**Table 3:** Correlations of Pearson values (*p* < 0.05) recorded between the analyzed variables during the period before, during, and after the shipwreck in the Port of Vila do Conde (Pará, Brazil). In italic: significant correlations

	pH	Temp	Cond.	TDS	Sal.	OD	Turb	STS	DBO	DQO	NO2
pH											
Temp	*− 0.175*]										
EC	− 0.406*	0.154									
TDS	− 0.462*	*0.576*	*0.739*								
Sal.	− 0.272*	0.03	*0.884*	0.524							
OD	*0.636*	**−** 0.712*	− 0.175	− 0.546	− 0.015						
Turb.	**−** 0.632*	0.079	0.161	0.359	− 0.196	− 0.568					
STS	− 0.469	0.104	− 0.123	0.261	− 0.291	**−** 0.588*	*0.805*				
DBO	0.204	0.39	− 0.095	− 0.148	− 0.118	− 0.2	− 0.208	− 0.315			
DQO	− 0.572	0.419	*0.614*	0.445	*0.606*	− 0.396	− 0.016	− 0.197	0.279		
NO_2_	− 0.307	**−** 0.694*	0.205	− 0.154	0.27	0.41	0.103	0	**−** 0.618*	0.128	
NO_3_	0.315	− 0.6	*0.07*	− 0.347	0.074	*0.846*	− 0.281	− 0.536	− 0.232	− 0.084	*0.615*
SO_4_	0.244	0.339	0.535	0.366	0.414	0.102	− 0.138	− 0.441	0.047	0.174	− 0.173
PT	− 0.47	0.288	0.135	0.446	0.214	− 0.431	− 0.059	0.114	− 0.1	0.386	− 0.026
NH_3_	**−** 0.639*	0.386	0.453	*0.773*	0.273	**−** 0.589*	0.502	0.527	− 0.494	0.363	0.128
Cla	− 0.133	0.161	0.014	− 0.039	0.169	0.028	− 0.456	− 0.445	0.39	*0.621*	0.102

*Negative correlations

*T*, temperature; *EC*, electric conductivity; *TDS*, total dissolved solids; *Sal*., salinity; *OD*, dissolved oxygen; *Turb*., turbidity; *STS*, total suspended solids; *DBO*, biochemical oxygen demand; *DQO*, chemical oxygen demand; *Cla*, chlorophyll-a

A canonical redundancy analysis (RDA) was conducted to investigate the responses of each species/group to the changes in the limnological variables. Of the environmental variables, only BOD, COD, and TDS were included in the regression model of the RDA, as the other variables did not significantly explain the proportion of the residual variance and were, therefore, excluded from the analysis.

**Fig. 4:**
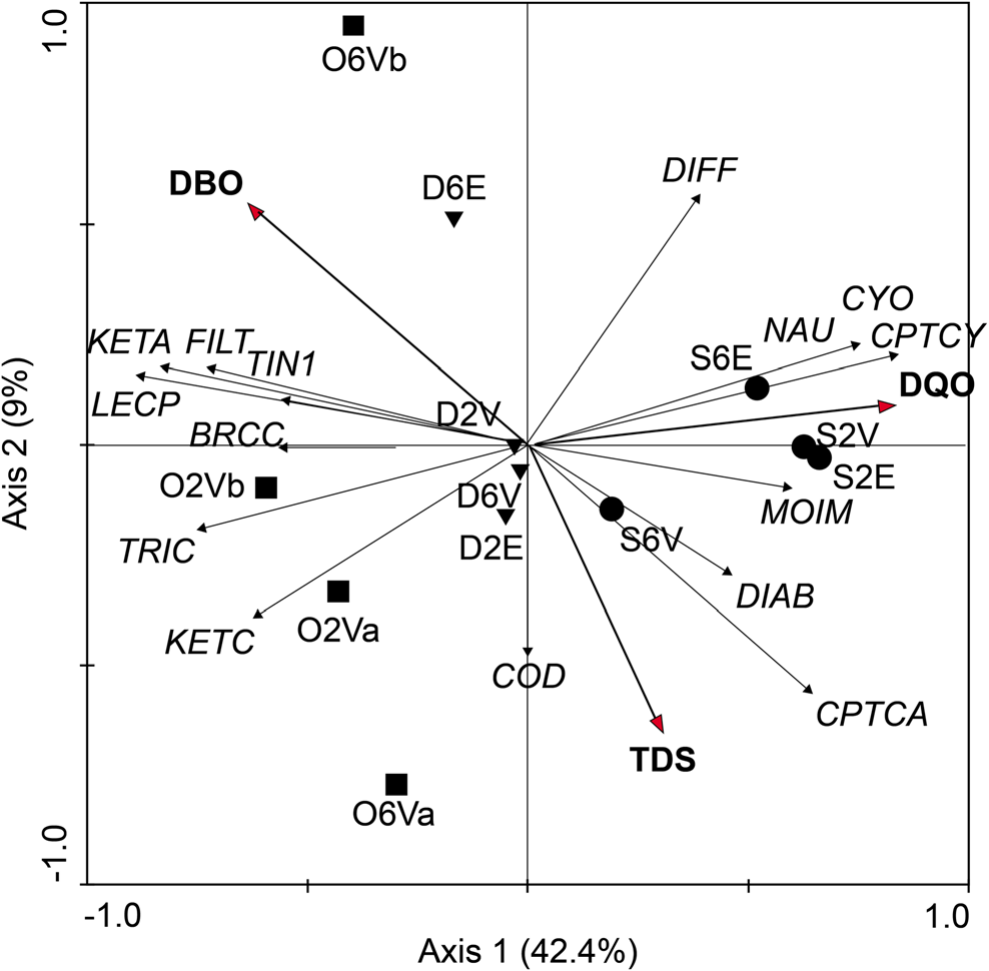
Canonical redundancy analysis (RDA) for the samples collected before (●), during (■), and after (▼) the shipwreck in the Port of Vila do Conde, State of Pará, Brazil. NAU: Nauplii, CPTCY: Copepodites (Cyclopoida), KETA: *Keratella americana*, CPTCA: Copepodites (Calanoida), TIN1: *Tintinnina* sp. 1, LECP: *Lecane proiecta*; CYO: *Cyclopoida* sp., FILT: *Filinia terminalis*, MOIM: *Moina minuta*, KETC: *Keratella cochlearis*, COD: *Codonella* sp. 1, DIAB: *Diaphanosoma birgei*, DIFF: *Difflugia* spp*.*, TRIC: *Trichocerca* sp. 1, BRCC: *Brachionus calyciflorus*

The correlation coefficients between the explanatory variables and the first two axes of the RDA are shown in Table 4. As shown in Fig. 4, axis 1 of the ordination explained 42.4% of the variation in the data. On this axis, the species/groups Cyclopoida (CYO), copepodites of Cyclopoida (CPTCY), and nauplii of Copepoda (NAU) were negatively correlated with the variables COD and TDS and with the samples collected before the shipwreck (in September 2015). In contrast, the projection of species and samples collected during and after the shipwreck was negatively correlated with BOD. Axis 2 explained 9% of the variance in the data and was positively correlated to species/groups with BOD, leading to a partial separation of the samples collected during and after the incidence.

**Table 4:** Correlation of environmental and species variables in axes 1 and 2, values, and *F* and *p* resulting from the RDA during the period before, during, and after the shipwreck in the Port of Vila do Conde (Pará, Brazil)

Variables	Abbreviation	Axis 1	Axis 2	*p* value	*F* ratio
Chemical oxygen demand	DQO	0.5689	− 0.1881	0.0039	4.66
Biochemical oxygen demand	BOD	− 0.1808	1.0000	0.0080	2.87
Total suspended solids	TDS	1.0000	–	0.0422	2.12

### Indicator species

We identified six species within the zooplankton community as significant indicator species (IndVal; Table 5). These species were selected using the specific characteristics of the different sampling periods and are detailed in this section. Group 1 contained samples collected before the shipwreck (September 2015) and was only represented by the species *Bosminopsis deitersi* (80%). Group 2 contained samples collected during the shipwreck and was also represented by a single species, *Trichocerca* sp. 1 (100%). Group 3 contained samples collected after the shipwreck and was represented by *L*. *proiecta* (56%), *D*. *birgei* (54%), *Tintinnina* species 1 (44%), and *K*. *americana* (41%).

**Table 5:** Zooplankton species with significant IndVal values with regard to the different periods studied: before (group 1), during (group 2), and after (group 3) the shipwreck in the Port of Vila do Conde (Pará, Brazil). In italic is the maximum IndVal value and *p* values ≤ 0.05

Species	Code	IndVal	Average	DP	*p*	Species association
*Bosminopsis deitersi*	BOSMDE	80	33.0	14.5	0.0253	Group 1
*Trichocerca* sp. 1	TRIRA	*100*	31.7	13.4	0.0063	Group 2
*Tintinnina* sp. 1	TINSP	44	38.5	2.2	0.0055	Group 3
*Lecane proiecta*	LECPR	56	38.2	10.3	0.0073	Group 3
*Keratella americana*	KERAM	41	36.6	1.7	0.0136	Group 3
*Diaphanosoma birgei*	DIABI	54	38.0	10.4	0.0188	Group 3

### Influence of the shipwreck on the zooplankton community

The community structure of zooplankton after the shipwreck differed to that observed in 2014 (September and December), mainly in terms of species composition, species richness, and the total density of organisms (Table 6). Despite the observed reduction in the density of the subclass Copepoda during the shipwreck, the early stages of nauplii and copepodites remained at high densities. However, we observed an increase (~ 11%) in members of the phylum Rotifera, which also contributed to the high densities of organisms, especially the genera *Keratella*, *Brachionus*, and *Trichocerca*. Finally, we observed a reduction of approximately 50% in plankton biomass (as measured by chlorophyll-a concentration) during the period of the shipwreck when compared with the values registered in September and December 2014 (Table 4).

**Table 6:** Assessment of the main biological parameters of the zooplankton community and chlorophyll-a, recorded in September to December 2014 and October 2015, in the Port area of Vila do Conde (State of Pará, Brazil)

	September–December 2014	Shipwreck period (October 2015)
Ameba testacea	9%	16%
Ciliophora	5%	5%
Cladocera	18%	14%
Copepod	14%	9%
Rotifera	36%	47%
Other	18%	9%
Species richness	22.25	17.44
Total density	4756 org./m^3^	1438 org./m^3^
Copepod	60%	54%
Rotifera	20%	37%
Diversity	2.91 bits/ind.	2.73 bits/ind.
Evenness	0.65	0.67
Chlorophyll-a	8.77	4.62

## Discussion

### Limnological variables

Dissolved oxygen (DO) is an important parameter in aquatic environments which facilitates the respiration of aerobic organisms (Baird 2002). Our results indicated that DO values were below the maximum allowed value reported by CONAMA (2005), while BOD levels were above this threshold. These findings might be related to the large input of biological material and the decomposition of oxen associated with the shipwreck. For instance, the release of excessive organic matter into a body of water might trigger the proliferation of microorganisms, thereby increasing total respiration. As a result, a higher quantity of oxygen is consumed, which might lead to the death of fish and other macrobiota (Fiorucci and Benedetti Filho 2005; Valente et al. 1997).

A high concentration of P_total_ in the study region that is above the threshold level might be related to the decomposition of high quantities of organic matter discharged into the environment. For instance, excess levels of P in a given body of water might lead to algal overgrowth and cause eutrophication (Von Sperling 2005). In addition, extremely high concentrations of some nutrients and high values of some limnological variables that are above threshold levels might be related to economic activities in the region (Paz et al. 2011).

### Zooplankton

The zooplankton community was mainly represented by the subclass Copepoda and by the phylum Rotifera throughout all sampling periods (before, during, and after the shipwreck). The dominance of these organisms is related to the fact that they favor eutrophic environments and feed on bacteria and protozoa (Souza-Pereira and Camargo 2004). The high density of the early stages of nauplii and copepodites could be considered an adaptive strategy to adverse conditions, as the production of many offspring ensures that, at least, a small portion of them reaches adulthood (Almeida et al. 2009).

Rotifers were also common in the study area, where high frequencies of occurrence and densities were mainly observed after the shipwreck. Therefore, these organisms might respond quickly to environmental changes, potentially serving as indicator species that are sensitive to changes in water quality (Marneffe et al. 1998; Matsumura-Tundisi and Tundisi 2005). Furthermore, the patterns of abundance and richness of these species are reflected in other levels of the aquatic food chain (Serafim-Júnior et al. 2005). According to Sládeck (1983), bacteria, small algae, flagellates, and detritus are the main items eaten by rotifers. In polluted water, the particles of suspended solids and colloids and the derivatives of the decomposition of organic material represent rich food sources for zooplankton. Thus, the quantity and quality of available food are important factors that potentially limit the composition of the zooplankton community, affecting the development and reproduction of these organisms (Santos et al. 2006; Sipaúba-Tavares and Bachion 2002).

Our results show that the species *Lecane* sp. 1, *Trichocerca* sp. 1, *K*. *cochlearis*, *F*. *opoliensis*, *L*. *proiecta*, *B*. *caudatus*, *M*. *minuta*, and *D*. *birgei* were very common during and after the shipwreck. The dominance of rotifer species, mainly belonging to the families Brachionidae, Lecanidae, and Trichocercidae, has also been observed in other tropical environments impacted by human activity (Attayde and Bozelli 1998; Paggi and José De Paggi 1990; Serafim-Júnior et al. 2003; Sousa et al. 2008). Sendacz et al. (2006) compared zooplankton abundance and biomass in environments with differing degrees of trophic abundance near the city of São Paulo and found that eutrophication stimulates the dominance of rotifer species. Bërzinš and Pejler (1989) studied 585 environments in Sweden and found that the dominance of rotifer species is related to conductivity, the levels of total P, and suspended solids, and that the species *K*. *cochlearis* and *Filinia longiseta* were correlated with eutrophic environments.

Costa et al. (2016a) evaluated a region close to our study area and observed higher values of the recorded species/organisms (149 species/organisms, compared with 84 in the present study). However, the present study noted a dominance of nauplii and copepodites and Rotifera from the families Brachionidae and Lecanidae. These results support those by other studies conducted in the Amazon region (Bozelli 1992; Costa et al. 2016a, b) and Brazil (Branco and Cavalcanti 1999; Branco and Senna 1996; Lucinda et al. 2004; Matsumura-Tundisi and Tundisi 2005; Negreiros et al. 2010; Pinto-Coelho 1998; Sendacz et al. 2006).

### Interactions between limnological variables and zooplankton

Under high-nutrient scenarios, the mean size of the zooplanktonic organisms generally decreases, as smaller species with simpler life cycles and more rapid rates of reproduction appear in the community (Gliwicz 1969; Odum 1969).

Using our calculations of TSI, we classified species as supereutrophic and eutrophic for different sampling seasons, during and after the shipwreck, mainly at site S6, where we also found a lower density of organisms. Of note, changes in the trophic state of a given body of water cause profound changes in the structure of the zooplankton community (Matsumura-Tundisi and Tundisi 2005) and in other species higher up in the food chain, such as plankton-eating fishes (Neto et al. 2014). In addition, environments with high degrees of trophic abundance exhibit increased biomass with a reduced number of species (Souza-Pereira and Camargo 2004). Our observations during the shipwreck supported this phenomenon, when the total density of organisms was reduced. In contrast, soon after the shipwreck, we observed an increase in the density of organisms, which might be related to an increased level of eutrophication, as the higher trophic state results in a greater availability of food, which, in turn, leads to an increase in zooplankton populations (Bonecker et al. 2007; Serafim-Júnior et al. 2010).

In the present study, the results of the RDA show that the patterns of variation in the zooplankton community were significantly and positively related to fluctuations in the conditions of the aquatic environment in the different periods analyzed. The three limnological variables BOD, COD, and TDS significantly explained the majority of variation in species composition/community groups of zooplankton. Our results showed that variation in these parameters were decisive in structuring the zooplankton community, particularly during the shipwreck.

The test of indicator species (IndVal) selected species according to the different periods analyzed. The species *L*. *proiecta*, *D*. *birgei*, *Tintinnina* sp. 1, and *K*. *americana* were effective indicator species of the trophic conditions following the shipwreck. Only *Trichocerca* sp. 1 was characteristic of the period during the shipwreck and could be used as an indicator of changes that took place at the time of the shipwreck. This species is a cosmopolitan and opportunistic organism that feeds on algae and typically occurs in changing environments with eutrophic conditions (Shiel and Koste 1992).

### Influence of the shipwreck on the zooplankton community

Our results showed that the shipwreck profoundly influenced the distribution and composition of the zooplankton community. Although most species co-exist under certain environmental conditions, certain organisms are limited by fluctuations in physicochemical factors, such as temperature, dissolved oxygen, salinity, and high concentrations of oil concentrations (Gannon and Stemberger 1978; Wake 2005).

We observed changes to the structure of the zooplankton community—especially with regard to the composition, density, and abundance of the species/groups—when comparing it against data from environmental monitoring in the region in 2014, especially with regard to the composition, density, and abundance of the species/groups (unpublished data). This observation was mainly related to a reduction in the subclass Copepoda in the order Cladocera, during the shipwreck. These were probably the only organisms that remained that had the most effective adaptive strategies to environmental modifications (Tundisi and Matsumura-Tundisi 2008).

Changes to the structure and composition of the community also indirectly affected the trophic level of the ecosystem. Such changes include the appearance of new species, an increase in the density of some organisms (e.g., nauplii, copepodites), and the disappearance of more sensitive species, all of which were observed after the shipwreck. However, other factors might have also influenced the dynamics of the community, such as competition with species that were better adapted to local conditions, predation, and parasitism. Competition for limited resources, in the case of major changes to the environment, is one of the main factors determining the diversity and species composition (Gannon and Stemberger 1978).

The results of the present study should be treated with caution, as they might not only be related to the impacts of the shipwreck but could also reflect local environmental conditions. For example, rainfall is a determining factor on the variation of some limnological parameters, which are constantly being modified by human activities. Furthermore, the study region exhibits peculiar dynamics. For instance, the River Pará, which is considered the largest river in the region, displays the characteristics of an estuary (Gregório and Mendes 2009). Snedaker and Getter (1985) claim that estuarine ecosystems have a strong natural ability to maintain and renew balance after a disturbance, as long as they retain the basic features of the habitat that favors the formation of this environment. However, processes that cause noticeable changes to the dominant patterns of an ecosystem affect the entire structure of the system.

## Conclusions

The environmental conditions of the region and ongoing environmental impacts have caused fluctuations in the structure of the zooplankton community in the River Pará, northern Brazil. The environment of the river is significantly impacted by the release of effluents and waste from various sources, particularly close to the port and industrial area. During the study period, the zooplankton community was mainly composed of organisms of the subclass Copepoda and the phylum Rotifera. The predominance of these organisms might be related to the large quantities of nutrients generated by the shipwreck. During the shipwreck, the composition of organisms declined, which might be associated with the large influx of nutrients and/or contaminants into the aquatic system.

One indicator of negative impacts on the aquatic ecosystem is the occurrence of many opportunistic species, which was observed in the present study. The rapid changes observed in the zooplankton community might be related to the possible impacts of the shipwreck and other local anthropogenic activities. Although local hydrodynamics allows for the constant renewal of this environment, we recommend the development of a continuous biomonitoring program using the zooplankton community as a tool to establish core strategies for the management and conservation of local biodiversity. This approach would result in the development of more effective public policies to control and mitigate anthropogenic activities in this region.

## References

[CR1] Almeida VLS, Dantas ÊW, Melo-Júnior M, Bittencourt-Oliveira MC, Moura AN (2009). Zooplanktonic community of six reservoirs in northeast Brazil. Braz J Biol.

[CR2] American Public Health Association (APHA) (2005). Standard methods for the examination of water and wastewater, 21st edn. American Public Health Association.

[CR3] Attayde JL, Bozelli RL (1998). Assessing the indicator properties of zooplankton assemblages to disturbance gradients by canonical correspondence analysis. Can J Fish Aquat Sci.

[CR4] Baird C (2002) Química Ambiental. Metais Pesados Tóxicos, 2nd edn. Ed. Bookman, Porto Alegre (in Portuguese)

[CR5] Bërzinš B, Pejler B (1989). Rotifers occurrence and trophic degree. Hydrobiologia.

[CR6] Bonecker CC, Nagae M, Bletter MCM, Velho LFM, Lansac-Tôha FA (2007). Zooplankton biomass in tropical reservoirs in Southern Brazil. Hydrobiologia.

[CR7] Bozelli RL (1992). Composition of the zooplankton community of Batata and Mussurá Lakes and of the Trombetas River, State of Pará, Brazil. Amazoniana.

[CR8] Branco CWC, Cavalcanti CGB (1999) A ecologia das comunidades planctônicas no lago Paranoá. In: Henry R (ed) Ecologia de reservatórios: estrutura, função e aspectos sociais. FAPESP/FUNDIBIO, Botucatu, pp 575–595 (in Portuguese)

[CR9] Branco CWC, Senna PAC (1996). Relations among heterotrophic bacteria, chlorophyll-a, total phytoplankton, total zooplankton and physical and chemical features in the Paranoá Reservoir, Brasília, Brazil. Hydrobiologia.

[CR10] Cairns J, McCormick PV, Niederlehner BR (1993). A proposal framework for developing indicators of ecosystem health. Hydrobiologia.

[CR11] CETESB – Companhia de Tecnologia e Saneamento Ambiental (1978). Determinação do zooplankton marinho: métodos qualitativos e quantitativos. Normatização técnica L5-301.

[CR12] CETESB – Companhia de Tecnologia e Saneamento Ambiental (2000). Zooplâncton de água doce: métodos qualitativo e quantitativo método de ensaio. Normalização técnica L5-304.

[CR13] CONAMA – Conselho Nacional do Meio Ambiente Resolução n° 357, de 17 de março de 2005 (2005) Ministério do Meio Ambiente, pp 23 (in Portuguese)

[CR14] Costa BNS, Pinheiro SCC, Amado LLA, Lima MO (2016). Microzooplankton as a bioindicator of environmental degradation in the Amazon. Ecol Indic.

[CR15] Costa BNS, Pinheiro SCC, Amado LLA, Lima MO (2016). Microzooplankton as an indicator of environmental quality at an industrial complex in the Brazilian Amazon. Ecol Indic.

[CR16] Dufrene M, Legendre P (1997) Species assemblages and indicator species: the need for a flexible asymmetrical approach. Ecol Monogr 67:345–366. https://doi.org/10.1890/0012-9615(1997)067[0345:SAAIST]2.0.CO;2

[CR17] Eskinazi-Sant’anna EM, Menezes R, Costa IS, Araújo M, Panosso R, Attayde JL (2013). Zooplankton assemblages in eutrophic reservoirs of the Brazilian semi-arid. Braz J Biol.

[CR18] Fiorucci AR, Benedetti Filho E (2005). A importância do oxigênio dissolvido em ecossistemas aquáticos. Quimica Nova na Escola.

[CR19] Gannon JE, Stemberger RS (1978). Zooplankton (especially crustaceans and rotifers) as indicators of water quality. T Am Microsc Soc.

[CR20] Gliwicz ZM (1969). Studies on the feeding of pelagic zooplankton in lakes with varying trophy. Ekologia Polska.

[CR21] Gregório AMS, Mendes AC (2009). Characterization of sedimentary deposits at the confluence of two tributaries of the Pará River estuary (Guajará Bay, Amazon). Cont Shelf Res.

[CR22] Hofmann RM (2015) Impactos Ambientais causados pelas obras de construção e ampliação de portos marítimos no Brasil com ênfase nas comunidades pesqueiras. Brasília-DF: Consultoria Legislativa da Câmara dos Deputados, Agosto de 2015. Disponível em: http://bd.camara.gov.br/bd/handle/bdcamara/26179 (in Portuguese). Accessed 12 Aug 2016

[CR23] IEC-SEMAN, Report No 028 (2015a) Relatório Técnico Referente aos Impactos Ambientais nas águas Superficiais ocasionados pelo Naufrágio de um Navio de Carga Viva ocorrido no Porto de Vila do Conde – Barcarena/Pará. pp 74 (in Portuguese)

[CR24] IEC-SEMAN, Report No 017 (2015b) Relatório Técnico Referente ao Monitoramento da Água Superficial da Área de Influência do Porto de Vila do Conde. pp 118 (in Portuguese)

[CR25] Lamparelli MC (2004) Grau de trofia em corpos d’água do estado de São Paulo: avaliação dos métodos de monitoramento. Tese (Doutorado) – Instituto de Biociências da Universidade de São Paulo, São Paulo. (in Portuguese)

[CR26] Legendre P, Birks HJB (2012). From classical to canonical ordination. Tracking environmental change using lake sediments.

[CR27] Legendre P, Legendre L (2012). Numerical ecology. 3rd English edition.

[CR28] Lucinda I, Moreno IH, Melão MGG, Matsumura-Tundisi T (2004). Rotifers in freshwater habitats in the upper Tietê River basin, São Paulo State, Brazil. Acta Limnologica Brasiliensia.

[CR29] Marneffe Y, Comblin S, Thome JP (1998). Ecological water quality assessment of the Butgenbach lake (Belgium) and its impact on the River Warche using rotifers as bioindicators. Rotifera VIII: A Comparative Approach.

[CR30] Matsumura-Tundisi T, Tundisi JG (2005). Plankton richness in a eutrophic reservoir (Barra Bonita Reservoir, SP, Brazil). Hydrobiologia.

[CR31] Negreiros NF, Santos-Wisniewski MJS, Santos RM, Rocha O (2010). The influence of environmental factors on the seasonal dynamics and composition of Rotifera in the Sapucaí River arm of Furnas Reservoir, MG, Brazil. Biota Neotropica.

[CR32] Neto AJG, Silva LC, Saggio AA, Rocha O (2014). Zooplankton communities as eutrophication bioindicators in tropical reservoir. Biota Neotropica.

[CR33] Odum EP (1969). The strategy of ecosystem development. Science.

[CR34] Paggi JC, José De Paggi S (1990). Zooplâncton de ambientes lóticos e lênticos do Rio Paraná médio. Acta Limnológica Brasiliensia.

[CR35] Park GS, Marshall HG (2000). Estuarine relationships between zooplankton community structure and trophic gradients. J Plankton Res.

[CR36] Paz AC, Frédou FL, Frédou T (2011). Caracterização da atividade pesqueira em Vila do Conde (Barcarena, Pará), no estuário amazônico. Boletim do Museu Paraense Emílio Goeldi. Ciências Humanas.

[CR37] Pinto-Coelho RM (1998). Effects of eutrophication on seasonal patterns of mesozooplankton in a tropical reservoir: a 4-year study in Pampulha Lake, Brazil. Freshw Biol.

[CR38] Pinto-Coelho RM, Bezerra-Neto JF, Morais CA (2005). Effects of eutrophication on size and biomass of crustacean zooplankton in a tropical reservoir. Braz J Biol.

[CR39] Piratoba ARA, Ribeiro HMC, Morales GP, Gonçales WG (2017) Caracterização de parâmetros de qualidade da água na área portuária de Barcarena, PA, Brasil. Revista Ambiente e Água 12 (in Portuguese)

[CR40] Porto MM, Teixeira SG (2002). Portos e Meio Ambiente.

[CR41] Rodrigues JEC, Szlafstein CF (2013). Análise do porto de vila do conde como uma área de ameaça potencial ao derramamento de óleo. Revista GeoAmazônia, Belém.

[CR42] Sampaio EV, Rocha O, Matsumura-Tundisi T, Tundisi JG (2002). Composition and abundance of zooplankton in the limnetic zone of the Paranapanema River, Brazil. Braz J Biol.

[CR43] Sant’anna JA (1998). Diagnóstico da infra-estrutura de transportes. In: Rede Básica de Transportes da Amazônia. IPEA, Brasília, Cap.

[CR44] Santos MAPF, Melão MGG, Lombardi AT (2006). Life history characteristics and production of *Ceriodaphnia silvestrii* Daday (Crustacea, Cladocera) under different experimental condition. Acta Limnologica Brasiliensia.

[CR45] Sendacz S, Caleffi S, Santos-Soares J (2006). Zooplankton biomass of reservoirs in different trophic conditions in the state of São Paulo, Brazil. Braz J Biol.

[CR46] Serafim-Júnior M, Bonecker CC, Rossa DC, Tôha FAL (2003). Rotifers of the upper Paraná River floodplain: additions to the checklist. Braz J Biol.

[CR47] Serafim-Júnior M, Ghidini AR, Neves GP, Brito L, Andreoli CV, Carneiro C (2005). Comunidade Zooplanctônica. Gestão integrada de mananciais de abastecimento eutrofizados.

[CR48] Serafim-Júnior M, Perbiche-Neves G, Brito L, Ghidini AR, Casanova SMC (2010). Variação espaço- temporal de Rotíferos em um reservatório eutrofizado no sul do Brasil. Iheringia série Zoologia.

[CR49] Shiel RJ, Koste W (1992). Rotifera from Australian inland waters VIII. Trichocercidae (Monogononta). Trans R Soc S Aust.

[CR50] Silva WM (2011). Potential use of Cyclopoida (Crustacea, Copepoda) as trophic state indicators in tropical reservoirs. Oecologia Brasiliensis.

[CR51] Sipaúba-Tavares LH, Bachion MA (2002). Population growth and development of two species of Cladocera, *Moina micrura* and *Diaphanosoma birgei*, in laboratory. Braz J Biol.

[CR52] Sládeck V (1983). Rotifers as indicators of water quality. Hydrobiologia.

[CR53] Snedaker SC, Getter CD (1985). Coasts: coastal resources management guidelines. Coastal Publication no. 2, Renewable Resources Information Series. U.S. Agency for International Development, and National Park Service.

[CR54] Sousa W, Attayde JL, Rocha E, Eskinazi-Sant’anna EM (2008). The response of zooplankton assemblages to variations in the water quality of four man-made lakes in semi-arid northeastern Brazil. J Plankton Res.

[CR55] Souza-Pereira PEE, Camargo AFM (2004). Efeito da salinidade e do esgoto sobre a comunidade zooplanctônica, com ênfase nos copépodes do estuário do Rio Itanhaém, estado de São Paulo, Acta Scientiarum. Biological Sciences, Maringá.

[CR56] Strickland JDH, Parsons TR (1972). A practical handbook of seawater analysis, 2nd edn, Bulletin 167.

[CR57] Tundisi JG, Matsumura-Tundisi T (2008). Limnologia.

[CR58] Valente JPS, Padilha PM, Silva AMM (1997) Oxigênio dissolvido (OD), Demanda Bioquímica de Oxigênio (DBO) e Demanda Química de Oxigênio (DQO) como parâmetros de poluição no Ribeirão Lavapés/Botucatu-SP. Eclética Química. pp 22 (in Portuguese) 10.1590/S0100-46701997000100005

[CR59] Von Sperling M (2005) Introdução à qualidade das águas e ao tratamento de esgotos, 3rd edn. Vol. 1. Belo Horizonte: Departamento de Engenharia Sanitária e Ambiental. Belo Horizonte: Ed. da UFMG (in Portuguese)

[CR60] Wake H (2005) Oil refineries: a review of their ecological impacts on the aquatic environment. Estuar Coast Shelf S:62, 131–140

